# Advancing lung organoids for COVID-19 research

**DOI:** 10.1242/dmm.049060

**Published:** 2021-06-28

**Authors:** Jelte van der Vaart, Mart M. Lamers, Bart L. Haagmans, Hans Clevers

**Affiliations:** 1 Oncode Institute, Hubrecht Institute, Royal Netherlands Academy of Arts and Sciences and University Medical Centre, Uppsalalaan 8, Utrecht 3584 CT, The Netherlands; 2 Viroscience Department, Erasmus University Medical Centre, Rotterdam 3015 GD, The Netherlands

## Abstract

The COVID-19 pandemic has emphasised the need to develop effective treatments to combat emerging viruses. Model systems that poorly represent a virus' cellular environment, however, may impede research and waste resources. Collaborations between cell biologists and virologists have led to the rapid development of representative organoid model systems to study severe acute respiratory syndrome coronavirus 2 (SARS-CoV-2). We believe that lung organoids, in particular, have advanced our understanding of SARS-CoV-2 pathogenesis, and have laid a foundation to study future pandemic viruses and develop effective treatments.

## Abstract (precis)

**Summary:** Pulmonary organoid model systems for COVID-19 research have played a significant role in understanding SARS-CoV-2 pathogenesis and may change the way we screen for potential antivirals in the future.

## Introduction

Severe acute respiratory syndrome coronavirus 2 (SARS-CoV-2) first emerged at the end of 2019, and has caused a pandemic of unprecedented scale and societal impact. The resulting disease, COVID-19, is characterised by pulmonary symptoms ranging from mild upper-airway disease to life-threatening acute respiratory distress syndrome (ARDS). With over 3.85 million deaths attributable to COVID-19 at the time of publication, this pandemic has underlined the importance of studying zoonotic viruses, preferably before they emerge as human pathogens. An important requirement to better understanding the pathogenesis of infectious diseases is the establishment of representative model systems. Because many of the viruses considered pandemic threats cause pulmonary disease, experimental platforms to study pulmonary infections are crucial. Historically, most *in vitro* coronavirus studies have been performed on two-dimensional (2D) cell lines like VeroE6, Caco-2 and Calu-3 cells (Takayama, 2020). Although these cell lines are often highly susceptible to these viruses, they may fail to model key aspects of the viral life cycle, and antiviral compounds that work *in vitro* may fail in patients (Hernandez et al., 2020; Wang et al., 2020). Cell lines are known for intrinsic abnormalities that might influence the viral replication cycle. For example, in VeroE6 cells, SARS-CoV-2 enters through the endosomal route after cleavage by cathepsins, whereas in primary airway cells, SARS-CoV-2 enters the cell at the plasma membrane after serine protease-mediated cleavage (Mykytyn et al., 2021). As a consequence, propagation of SARS-CoV-2 leads to the introduction of cell culture-adaptive mutations in the spike protein multibasic cleavage site (Klimstra et al., 2020; Lamers et al., 2021b; Lau et al., 2020; Liu et al., 2020; Ogando et al., 2020). These adapted viruses do not behave like authentic SARS-CoV-2, as they are less pathogenic (Johnson et al., 2021; Lau et al., 2020) and do not transmit (Peacock et al., 2021). In addition to cell lines, multiple animal models have been used to study COVID-19. Whereas mice are not susceptible to wild-type SARS-CoV-2 infection (Zhou et al., 2020b), animals that overexpress the human variant of angiotensin converting enzyme 2 (ACE2) show high viral loads, not only in the lungs but also in the brain (Sun et al., 2020). In golden Syrian hamster, SARS-CoV-2 can use hamster ACE2 for entry, although the animals do not develop severe lung disease upon viral infection as seen in hospitalised COVID-19 patients (Chan et al., 2020). Similarly, in ferrets, the disease is relatively mild, and virus replication is mainly observed in the upper respiratory tract (Shi et al., 2020). In addition, SARS-CoV-2 rapidly acquires spike protein mutations in ferrets as a result of critical species-specific differences in ACE2 receptor interactions (Richard et al., 2020), showing the complexity of the response to viral infection in different animal hosts (Leist et al., 2020; Rosa et al., 2021). All these models are costly and require specialised animal BSL-3 facilities. Over the past year, human stem cell-derived organoids have emerged as powerful tools for COVID-19 research, bridging the gap between cell lines and *in vivo* animal models.

“Over the past year, human stem cell-derived organoids have emerged as powerful tools for COVID-19 research, bridging the gap between cell lines and *in vivo* animal models.”

## Recap of current airway organoid technologies

Organoids are three-dimensional (3D) *in vitro* structures grown from stem cells, which consist of organ-specific cell types that self-organise through cell sorting and spatially restricted lineage commitment (Clevers, 2016; Lancaster and Knoblich, 2014). Organoids can be established from two distinct stem cell populations: adult stem cells (ASCs) derived from adult or foetal tissue, or pluripotent stem cells (PSCs) [induced PSCs (iPSCs) and embryonic stem cells (ESCs)]. Under optimal conditions, a comprehensive set of cell types from the tissue of interest is generated. Both stem cell types have been employed to generate lung organoids. Yet, the derivation of airway organoids and their subsequent characteristics differ greatly between the two stem cell types. iPSC-derived organoids are formed by generating 3D aggregates from the pertinent iPSCs. The subsequent lung fate specification is accomplished by culturing the aggregates in a series of growth factor-enriched media designed to mimic the journey of an embryonic stem cell on its way to building lung tissues in a developing embryo. By contrast, ASC-derived organoids are generated by isolation of resident stem cells from the airways by providing a 3D matrix as well as a single growth factor cocktail (van der Vaart and Clevers, 2020).

Differentiation of iPSCs to airway epithelium generates structures that consist of multiple airway cell types, including basal, club and ciliated cells (Dye et al., 2015; Firth et al., 2014; Huang et al., 2014; Konishi et al., 2016; McCauley et al., 2017; McIntyre et al., 2014; Mou et al., 2012; Wilkinson et al., 2017), as well as alveolar cell types: alveolar type I and II (ATI and ATII) cells (van Riet et al., 2020). These iPSC models may also contain non-epithelial/non-endodermal elements including mesenchyme and endothelium. Although iPSC-derived lung progenitors can be induced to form 3D alveolar structures with ATI and ATII cells (de Carvalho et al., 2019), the combination of airway and alveoli has yet to be achieved. Similar challenges existed for ASC-derived human lung organoids, with initial cultures containing the major airway cell types, whereas alveolar cells were absent (Sachs et al., 2019). Against this backdrop, we highlight that the COVID-19 pandemic has fuelled the development of multiple novel, long-term lung culture systems.

“When SARS-CoV-2 emerged in December 2019 in Wuhan, China, respiratory models were rapidly employed to study the infection of this newly identified coronavirus.”

## Airway organoids as tools for studying COVID-19

Organoids have been used as models of a multitude of pathologies, including infectious diseases (Clevers, 2016; Lancaster and Huch, 2019). Human intestinal organoids from ASCs were the first to allow successful *in vitro* propagation of human noroviruses (Estes et al., 2019; Ettayebi et al., 2016; Haga et al., 2020). During the Zika virus outbreak, iPSC-derived cerebral organoids allowed the fast discovery of viral replication in dividing neural progenitors and subsequent demise of the latter, explaining how foetal, but not adult, brains could be affected by the virus (Qian et al., 2017; Watanabe et al., 2017). Furthermore, ASC-derived human oral mucosal organoids were infectible by herpes simplex virus and human papilloma virus (Driehuis et al., 2019). Importantly, airway organoids have been used to study a broad range of viruses, including respiratory syncytial virus and influenza virus (Sachs et al., 2019; Zhou et al., 2018).

When SARS-CoV-2 emerged in December 2019 in Wuhan, China, respiratory models were rapidly employed to study the infection of this newly identified coronavirus. ASC-derived airway organoids were infected as air–liquid interface (ALI) cultures (Lamers et al., 2020, 2021b) and as 3D structures (Salahudeen et al., 2020; Suzuki et al., 2020 preprint). Lamers and colleagues showed essential viral life-cycle stages, which include intracellular double-vesicle membranes. Studies on 2D ALI cultures identified ciliated cells as a primary target of SARS-CoV-2, but also noted occasional infection of club cells (Lamers et al., 2020, 2021a). Three-dimensional cultures, however, showed primary infection in club cells (Salahudeen et al., 2020) or basal cells (Suzuki et al., 2020 preprint), indicating that growth conditions, differentiation status or perhaps differences between donors affect viral tropism. The potential for using ASC-derived airway cultures for virus propagation of SARS-CoV-2 was shown by Lamers et al. (2021b). Most importantly, cell culture-adaptive mutations in the multibasic cleavage site of the spike protein of SARS-CoV-2, which mediates viral entry into the host cell, typically seen upon propagation of the virus in cell lines, did not occur in viruses propagated on these ALI cultures of airway organoids. This indicated that this culture system accurately models viral target cells *in vivo* (Lamers et al., 2021b). Moreover, fundamental virology studies can be aided by the use of this representative model system. This is evidenced by the discovery that SARS-CoV-2 enters human airway cells via serine protease-mediated entry and not via endocytosis/cathepsin-mediated entry, as is the case in VeroE6 cells and other cell lines commonly used in virology laboratories (Hoffmann et al., 2020; Mykytyn et al., 2021). The latter observation explains why the endocytosis pathway inhibitor (hydroxy-)chloroquine emerged from cell line screens, yet was ineffective in the clinic (Hernandez et al., 2020). A limitation, however, remains that these ALI cultures do not allow modelling of the immune system, as the basal side of the cells is attached to plastic and therefore inaccessible to immune cells. Three-dimensional models that can be exposed to direct cell–cell interaction at their basal side might overcome this limitation. These future models will be valuable in studying immune responses towards virally infected cells and could potentially be used to find new immune modulators that limit disease severity in patients.

## Alveolar organoid systems as potential models for ARDS

Although these airway models recapitulate some findings in COVID-19 patients, the search for genuine alveolar model systems remained. Studying alveolar response to SARS-CoV-2 infection is critical as most hospitalised COVID-19 patients are admitted due to ARDS, but freshly isolated primary alveolar cultures from healthy individuals were found only minimally susceptible to SARS-CoV-2 (Hou et al., 2020). In addition, human ATII cells rapidly differentiate to ATI-like cells in 2D cell culture, limiting the possibilities to study ATII biology (Bove et al., 2014). Several studies used existing iPSC-derived cells, first described in 2017 (Jacob et al., 2017), to generate alveolar epithelium (Jacob et al., 2019). This differentiation was extended to specific ATII cells by Abo and colleagues. The authors induced iPSCs to differentiate towards ATII (iATII) cells, which led to the expression of the main SARS-CoV-2 viral entry factors ACE2 and transmembrane serine protease 2 (TMPRSS2), comparable to levels in freshly isolated ATII cells (Abo et al., 2020 preprint). These ALI cultures were thereafter permissive to SARS-CoV-2 infection. The expression of these vital viral entry factors was in line with the expression *in vivo* in ATII and ciliated cells in the lung (Hikmet et al., 2020; Jia et al., 2005; Qi et al., 2020). The infection could be blocked by the antiviral drug remdesivir and by TMPRRS2 inhibition, while a cathepsin B/L inhibitor that showed effect in VeroE6 cells did not block virus replication in iATII cells (Huang et al., 2020). iPSC-derived alveolar model systems also revealed other US Food and Drug Administration (FDA)-approved drugs to have potential in inhibiting SARS-CoV-2 infection, including the tyrosine kinase inhibitor imatinib, the immunosuppressant mycophenolic acid and the antimalarial agent quinacrine dihydrochloride (Han et al., 2021). Although the effects of these drugs remain to be investigated in patients, these studies show the potential of iPSC-derived alveolar model systems as pre-clinical models over standardised immortalised cell lines.

Lamers and colleagues applied a different approach with human foetal lung epithelial stem cells to achieve alveolar differentiation (Lamers et al., 2021a), using lung bud tip organoids described earlier (Nikolić et al., 2017). These foetal lung bud tip organoids displayed alveolar differentiation potential in 3D (Nikolić et al., 2017) and were used to establish ALI cultures in combination with foetal fibroblasts to differentiate into bronchioalveolar-like cultures. These cultures were composed of both alveolar-like and bronchiolar-like areas (Lamers et al., 2021a). This mix of cell types allows studying interactions between multiple areas in the airways, thereby providing a more complete view compared to studying a single area. A similar combinatorial culture system was recently established by using iPSCs (Rodrigues Toste de Carvalho et al., 2021; Tiwari et al., 2021). The susceptibility of these optimised foetal or iPSC-derived ALI cultures to SARS-CoV-2 infection appears to be higher than that of the ASC 3D systems, which could be related to the facile access of the virus to the apical side of the cells or their differentiation status.

COVID-19 also motivated the development of several alveolar model systems derived from ASCs. These studies used primary ATII cells as starting material to generate 3D spheres that were composed of ATII cells only, while maintaining the potential to differentiate to ATI cells. Growth conditions and susceptibility to SARS-CoV-2 infection varied between studies (Katsura et al., 2020; Salahudeen et al., 2020; Youk et al., 2020). The system of Youk and colleagues (Youk et al., 2020) appears to be most permissive to SARS-CoV-2 infection, achieving infectious virus titres up to 10^4^ infectious units/ml (10^3^-fold change compared with input). Very similar ATII culture conditions were recently used by Lamers and colleagues (Lamers et al., 2021c preprint), resulting in similar infectious virus titres (using 3D and 2D ATII cultures). Katsura, Youk and Lamers et al. all reported the induction of interferon (IFN)-specific gene responses, attributed to type I and III IFNs, and a decrease in the expression of surfactant proteins (e.g. SFTPB, SFTPC and SFTPD). Other ATII markers did not exhibit decreased expression upon SARS-CoV-2 infection (Katsura et al., 2020; Lamers et al., 2021c preprint; Youk et al., 2020). Lower levels of surfactant proteins are detrimental to oxygen exchange, as these proteins are crucial in preventing alveolar collapse. They are also involved in innate and adaptive immune responses (Crouch and Wright, 2001; McCormack and Whitsett, 2002). In addition, Katsura and colleagues report the prominent expression of chemokines (CXCL10, CXCL11 and CXCL17) and cell death-related genes (*TNFSF10*, *CASP1*, *CASP4*, *CASP5* and *CASP7*) (Katsura et al., 2020). Overall, it seems that alveolar infection is characterised by type I/III IFN responses, proinflammatory responses (e.g. chemokines), apoptosis and low surfactant production. These responses are induced solely by epithelial cells, and future studies may investigate the influence of other lung cell types on these responses.

Together, the toolbox of pulmonary organoid model systems has increased rapidly over the past year. In our opinion, it now provides a more physiological experimental setting to study SARS-CoV-2 and other pathogens that target the lungs, be it airways or alveoli.

“It is evident that advancing the development of pulmonary organoid culture models has contributed to understanding pathogenesis and advancing drug development for SARS-CoV-2.”

## Future perspectives

**Fig. 1.**
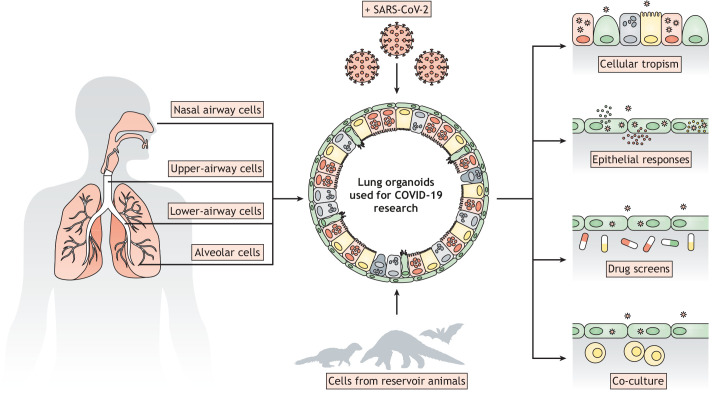
Overview of the potential of region-restricted lung organoids in virology.

As discussed above, the emergence of COVID-19 has boosted the establishment of many new lung organoid models, as well as revived older approaches. The extensive variation in protocols indicates that there may exist multiple ways to achieve our common goal, and further improvements to the current technologies are to be expected. Combining cell biology and virology expertise has already led to the development of applicable, representative and easily infectible model systems. Knowledge of stem cell biology combined with that of pulmonary development thus allows the generation of multiple organoid-based respiratory cell cultures of airways and alveoli (Fig. 1).

Organoid cultures originating from different parts of the respiratory tract could allow further insight into virus tropism, not only of SARS-CoV-2 but also other respiratory viruses. Cells of the upper airway could be used to study virus shedding or the role of host factors in the context of virus transmission, but might be less relevant when studying severe COVID-19, where infection of alveolar cells or inflammation in the distal lung can lead to potentially fatal ARDS (Ackermann et al., 2020; McGonagle et al., 2021; Menter et al., 2020). Alveolar models can be used to study the latter complication, either alone or in combination with other cell types, such as endothelial, stromal or immune cells (Fig. 1). Airway cells of the bronchus and bronchiole could be used to study local host responses and dissemination to the alveolus. It is vitally important to understand regional differences by using multiple model systems, which will further pinpoint the direct causes of disease severity and potentially elucidate effective therapies to alleviate this.

SARS-CoV-2, like many other human viruses, originates from animals and has presumably undergone adaptations when infecting humans. Organoids derived from reservoir animals could be used to culture and study viruses that have not yet been shown to infect humans but may do so in the future. The potential of these organoids has already been shown using bat- and feline-derived intestinal organoids to study coronaviruses (Tekes et al., 2020; Zhou et al., 2020a). Expanding these studies may reveal the adaptations required for viruses to infect other species (Fig. 1).

Several reports have shown the relationship between host genetic variants and susceptibility to SARS-CoV-2 and disease severity (Mohammadpour et al., 2021). The underlying biology can be studied in relevant organoid models derived from individuals with representative genetic backgrounds. The use of sophisticated genetic editing tools can also increase understanding of SARS-CoV-2 biology (Fig. 1). Genome-wide CRISPR-Cas9 screens have already identified a number of host factors like ACE2 that play a crucial role in SARS-CoV-2 infection (Wang et al., 2021; Wei et al., 2021). These studies were performed on classical cell lines (and have revealed a role for endocytosis in VeroE6 cells; see above), but we emphasise that these finding should be confirmed in more physiologically relevant models.

Contrarily, variants of SARS-CoV-2 that are more transmissible or escape from immunity are increasingly being identified (Plante et al., 2021). Organoids can potentially be used to identify the differences between these strains of SARS-CoV-2. Infection of the British variant (B.1.1.7) in airway, alveolar and intestinal organoids produced higher levels of viral particles late in infection compared to the ancestral strain (Lamers et al., 2021c preprint), identifying extended shedding as an *in vitro* correlate of viral fitness. The British variant also outcompeted the ancestor in airway organoids when both viruses were added to the same culture. The rapid comparison of SARS-CoV-2 variants in organoid systems may ultimately inform public health decision making.

Organoids have also emerged as a promising tool for predicting drug efficacy through high-throughput screens (Fig. 1). Norovirus infection in intestinal organoids could be blocked by a variety of antibodies (Alvarado et al., 2018). Moreover, iPSC-derived cerebral organoids have provided a platform for identifying treatments that limit neural progenitor cell death after Zika virus infection (Qian et al., 2017; Watanabe et al., 2017). In the past year, intestinal and airway organoid models have been implicated in predicting SARS-CoV-2 treatment discovery. Application of 25-hydroxycholesterol, remdesivir, IFN-λ or camostat decreases SARS-CoV-2 spread in the organoids (Krüger et al., 2021; Lamers et al., 2021a; Mykytyn et al., 2021; Suzuki et al., 2020 preprint; Zu et al., 2020). While these studies used known antiviral drugs or drugs based on known SARS-CoV-2 biology, a recent study employed iPSC-derived airway organoids in a 384-well format to uncover FDA-approved drugs that effectively lower viral replication (Han et al., 2020 preprint). A separate study used human iPSC-derived colorectal infected with SARS-CoV-2 organoids in a similar set-up and tested more than 1000 drugs (Duan et al., 2020). Many of these hits overlapped with those in lung organoids, indicating effective viral blockade in at least two epithelial tissues.

It is evident that advancing the development of pulmonary organoid culture models has contributed to understanding pathogenesis and advancing drug development for SARS-CoV-2. Continuing this progress will be essential to prepare for future pandemics.
